# Optimal cutoff of pretreatment neutrophil-to-lymphocyte ratio in head and neck cancer patients: a meta-analysis and validation study

**DOI:** 10.1186/s12885-018-4876-6

**Published:** 2018-10-11

**Authors:** Jae-Keun Cho, Myoung Woo Kim, Ick Soo Choi, Uk Yeol Moon, Min-Ji Kim, Insuk Sohn, Seonwoo Kim, Han-Sin Jeong

**Affiliations:** 1 Department of Otorhinolaryngology-Head and Neck Surgery, Inje University Ilsan Paik Hospital, Inje University School of Medicine, Goyang, Republic of Korea; 2 Department of Otorhinolaryngology-Head and Neck Surgery, Samsung Medical Center, Sungkyunkwan University School of Medicine, Seoul, 06351 Republic of Korea; 3 Statistics and Data Center, Research Institute for Future Medicine, Samsung Medical Center, Seoul, Republic of Korea

**Keywords:** Head and neck cancer, Inflammatory marker, Neutrophil, Lymphocyte, Outcomes, Prognosis

## Abstract

**Background:**

The prognostic role of neutrophil-to-lymphocyte ratio (NLR) has been proposed in head and neck squamous cell carcinoma (HNSCC). However, it is currently unclear which cutoff values of NLR could consistently and independently differentiate HNSCC patients to better and worse prognosis groups.

**Methods:**

We performed a meta-analysis of prognostic significance of pretreatment NLR values, using data extracted from 24 relevant articles. Main outcomes were overall survival (OS) and disease-free survival (DFS) in HNSCC patients. Pooled hazard ratio (HR) and 95% confidence intervals (95%CI) were calculated using the random effect model for outcomes. Impacts of NLR cutoff values across the studies were assessed with a meta-regression analysis. Results were validated using an independent data set of patients (*n* = 540).

**Results:**

Pretreatment high NLR values above the cutoff were significantly associated with shorter OS (HR = 1.96, 95%CI = 1.66–2.31) and DFS (HR = 1.90, 95%CI = 1.41–2.54). Of note, NLR cutoffs ranging from 1.9 to 6.0 did not affect HR of OS or DFS in meta-regression analyses. In an independent cohort, any NLR cutoff between 2 and 6 produced significant HR of OS, similarly. Instead of binary cutoffs, three subgroups of NLR (< 2, 2 to 6, and ≥ 6) showed significant differences of OS in survival analyses.

**Conclusions:**

Meta-analyses confirmed that pretreatment NLR values above the cutoff were associated with shorter survival in HNSCC patients. However, the binary cutoffs of NLR values were variable across studies. Rather, pretreatment NLR values below 2 and above 6 using a three-tier classification (< 2, 2 to 6, and ≥ 6) could consistently imply better and worse prognosis in HNSCC patients, which could be readily translated to clinics.

**Electronic supplementary material:**

The online version of this article (10.1186/s12885-018-4876-6) contains supplementary material, which is available to authorized users.

## Background

Systemic inflammation has proven to be a major contributing factor in cancer development and progression across a number of tumor types [1–4]. Biologically, tumor associated inflammatory response is recognized as one of cancer hallmarks [5]. High degree of systemic inflammation is associated with worse outcomes in cancer patients; meanwhile local inflammation with the infiltration of various immune cells around tumors reflects better outcomes [6–9]. As surrogate markers of systemic inflammation, serum albumin, C-reactive protein, neutrophil- and platelet-to-lymphocyte ratios have been investigated widely [10–13].

Systemic inflammation could have a prognostic value in head and neck squamous cell carcinoma (HNSCC) as in other cancers [4]. Neutrophil-to-lymphocyte ratio (NLR) (dividing the absolute neutrophil count by the absolute lymphocyte count) is one of various biomarkers of systemic inflammation. There have been numerous studies on the prognostic role of NLR in various selected populations of HNSCC [14–17]. One clinical advantage using NLR value to estimate prognosis is that it is a simple and readily measurable indicator from routine blood sampling of patients at diagnosis.

However, even with a wealth of reports about the prognostic role of NLR in HNSCC, most studies have adopted cutoff values of NLR driven by internal prognosis grouping without external validation [14–19]. Recent meta-analyses also confirmed the prognostic role of pretreatment NLR value in HNSCC, without suggesting the optimal cutoff value [20–23]. It is currently unclear which cutoff value of NLR could consistently and independently differentiate better and worse prognosis groups in HNSCC patients. Thus, this work was designed to explore whether pre-treatment NLR in HNSCC might have a prognostic significance across multiple studies (meta-analyses), the optimal cutoff values of NLR for consistent differentiation of HNSCC prognosis (evaluation of cutoff point), and whether such cutoff values might be still valid in an independent data set of HNSCC patients (external validation). Through these sequential analyses, we aimed to build a bridge between clinical findings and general application of NLR value in HNSCC.

## Methods

### Search strategy

This meta-analysis was performed according to the recommendations of the Preferred Reporting Items for Systematic Reviews and Meta-Analyses (PRISMA) 2009 guidelines [24]. We systematically searched the MEDLINE, EMBASE and Cochrane library databases focusing on clinical studies published prior to April 30th, 2017. Potentially relevant studies were identified using the following key words: [neutrophil lymphocyte ratio], in combination with [head and neck], [cancer], and [prognosis] (Additional file 1: Table S1). In addition, reference lists of retrieved articles were screened manually to identify additional eligible studies. No language restriction was imposed. Eligibility of these studies was decided through comprehensive reviews and discussions with multiple researchers.

### Study selection

Inclusion criteria for these studies were as follows: (i) enrolled patients had a histological diagnosis of HNSCC, (ii) pre-treatment NLR was calculated from peripheral blood samples before any treatments, and (iii) information about clinical outcomes (disease recurrence, metastatic tumor progression and death from any cause) was available (disease-free survival DFS, progression-free survival PFS, overall survival OS). Studies were excluded if there was insufficient information to calculate the hazard ratio (HR) and 95% confidence interval (95%CI) of outcomes.

### Data extraction and quality assessment

Two authors (JKC, HSJ) independently identified eligible articles and collected the following data: (i) publication information; the first author’s name, year of publication, country of study conducted, (ii) pre-treatment NLR data, (iii) clinical features: total patient numbers, TNM stages at diagnosis, disease outcomes and follow-up duration. Any disagreement was resolved by discussion. The Newcastle-Ottawa quality assessment scale for non-randomized studies was implemented to evaluate the quality of included studies (Additional file 1: Table S2) [25].

### Meta-analysis for prognostic impacts of pretreatment NLR status

NLR status, a binary variable (high NLR and low NLR), was defined according to the study-specific NLR cutoff value. To determine the effect of NLR status on clinical outcomes, HRs were pooled using the random effect model [26, 27]. HR of more than 1 indicated worse outcome for the group having NLR above cutoffs compared to the group having NLR below cutoffs. The precision of estimates was quantified by 95%CI.

Heterogeneity was measured by Higgins and Green I^2^ test [27]. Values of I^2^ ranged between 0% (no heterogeneity) and 100% (maximal heterogeneity). Heterogeneity of the study was considered to be substantial at *P* < 0.1 and I^2^ > 50%. We also evaluated potential publication bias with Egger’s regression test and funnel plot [26]. A sensitivity analysis with a trim-and-fill method was conducted. All above analyses were executed using R 3.3.2 (Vienna, Austria; http://www.r-project.org/) with a package of metafor. A two-sided *P* value of less than 0.05 was considered statistically significant.

### Meta-regression of cutoff values of NLR

Study-specific NLR cutoff values ranged from 1.9 to 6.0 (median = 3). Meta-regression analyses were then performed to determine whether the impact of NLR status on clinical outcomes was different according to each study-specific factor: the cutoff value of NLR, age, gender, tumor stage (I/II versus III/IV), tumor subsite (oral cavity, pharynx, larynx, others) and number of index tumors (single versus multiple). Results are presented as change of HR of OS and DFS.

### Validation study using an independent data set

To confirm the prognostic impact of NLR cutoff values estimated from previous analyses, we conducted a validation study using an independent cohort in our institution. All HNSCC patients were enrolled prospectively into our head and neck cancer registry and they provided written informed consents for use of their clinical and biological data under an Institutional Review Board approved protocol. From registered HNSCC patients, we included patients who had been treated for their HNSCC (newly diagnosed) between 2010 and 2014 (*n* = 540).(Additional file 2: Raw data for a validation study) The clinical characteristics of our cohort were comparable to the study patients enrolled in the meta-analyses (Additional file 1: Table S3). All patients followed the current standard treatment protocols (The National Comprehensive Cancer Network guideline, http://www.nccn.org). Patients with secondary cancers, other pathologies, or palliative treatments were excluded. HR on outcomes (OS and DFS) of NLR status according to possible NLR cutoffs in the range of 2 to 6 by increment of 0.1 were calculated in multivariate Cox proportional hazard regression analyses. The present study focused on pretreatment NLR values and clinical outcomes was approved by our Institutional Review Board again before data collection.

## Results

### Characteristics of identified studies

**Fig. 1:**
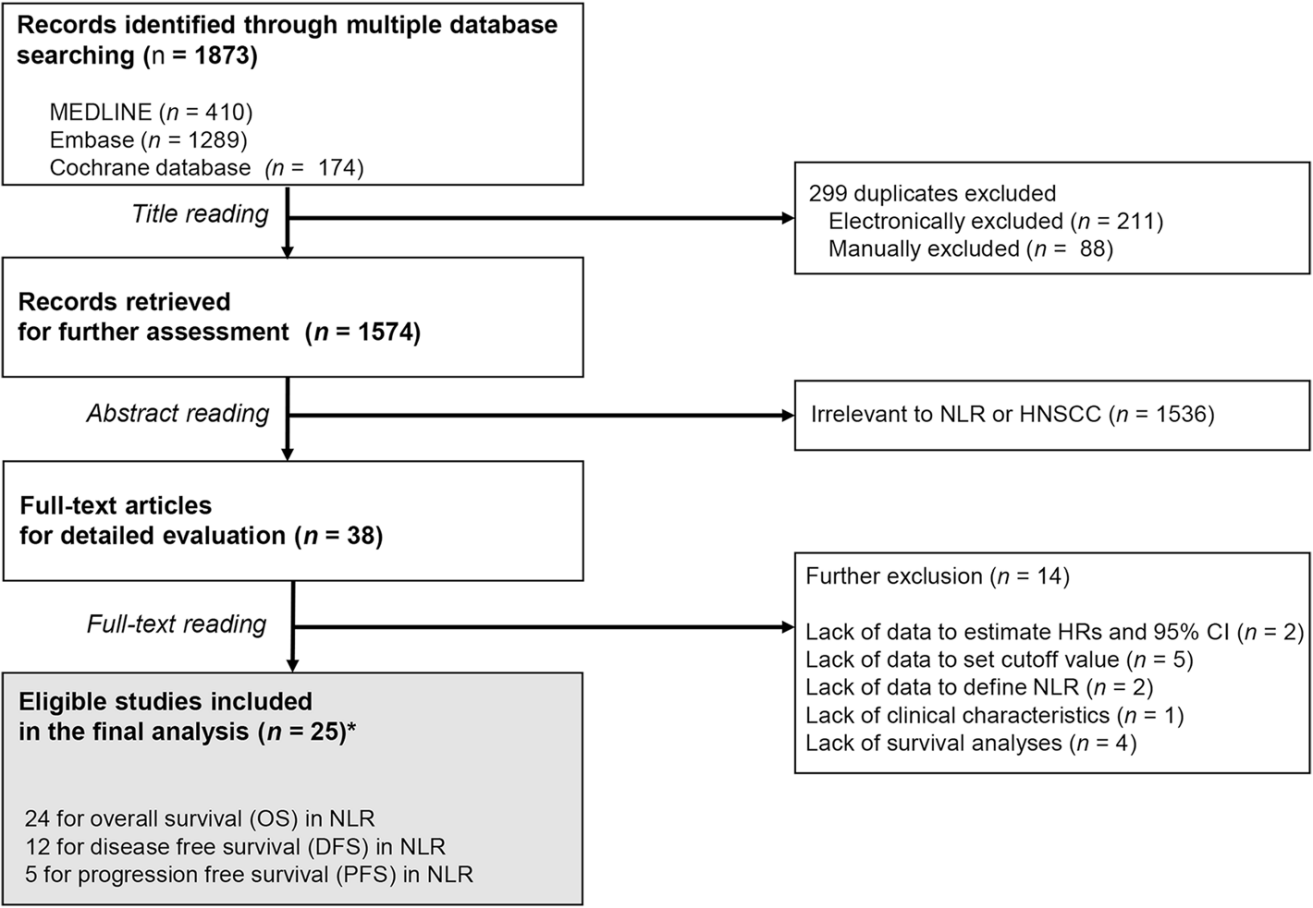
Flow chart of study selection process (*25 studies in 24 articles)

We identified 38 potentially relevant articles through multiple database searches. Among them, 14 were further excluded, mainly due to inability to estimate HR and 95%CI of outcomes. The article by Charles et al. [14] presented results with oropharyngeal cancer and non-oropharyngeal cancer separately. We regarded their study results as two independent studies. Thus, 25 observational studies in 24 articles were included in our meta-analysis (Fig. 1). Characteristics of included studies are listed in Table 1. Among them, 24 reported HRs for OS outcomes, including 12 and 5 studies for HRs of DFS and PFS, respectively.

**Table 1:** Characteristics of studies included in the final analyses

	Study (First Author)	Publication year	No. of subject	NLR cutoff	Stage	Index of tumor	Follow-up (mo)	Outcome	HR extraction	Multivariate adjustment
1	He [28]	2012	1410	2.17	I-IV	Pharynx^a^	41	OS, PFS	Reported	Yes
2	Millrud [29]	2012	20	6	I-IV	Others^b^	24	OS	Reported	No
3	Fang [30]	2013	226	2.44	I-IV	Oral cavity		OS, DFS	Reported	No
4	Rassouli [18]	2013	273	4.27		Others	45	DFS	Reported	No
5	Jin [31]	2014	229	3.6	III, IV	Pharynx		OS	Reported	Yes
6	Young [32]	2014	249	5		Pharynx	46	OS	Reported	No
7	Haddad [33]	2015	46	5	III, IV	Others	34	OS, DFS	Reported	No
8	Rachidi [34]	2015	543	4.39	I-IV	Others	64	OS	Reported	Yes
9	Salim [35]	2015	79	2.93	I-IV	Others		OS, PFS	Reported	No
10	Selzer [36]	2015	170	5	I-IV	Others		OS	Reported	Yes
11	Song [37]	2015	146	2.3		Pharynx	26	OS	Reported	No
12	Sun [19]	2015	251	2.7	I-IV	Pharynx	50	OS, PFS	Reported	Yes
13	Tu [16]	2015	141	2.17	I-IV	Larynx	51	OS, DFS	Reported	Yes
14	Charles [14] (1)^c^	2016	76	5	I-IV	Pharynx	29	OS, DFS	Reported	Yes
15	Charles [14] (2)^c^	2016	69	5	I-IV	Others	29	OS, DFS	Reported	Yes
16	Chua [38]	2016	380	3	I-IV	Pharynx		OS, DFS	Reported	Yes
17	Fu [39]	2016	420	2.59	III, IV	Larynx		OS	Reported	Yes
18	Ikeguchi [40]	2016	59	5	III, IV	Pharynx	38	OS	Reported	Yes
19	Kano [41]	2016	285	1.92	I-IV	Others	63	OS, DFS	Reported	Yes
20	Kim [42]	2016	104	3	III, IV	Others	39	OS, DFS	Reported	Yes
21	Moon [43]	2016	153	3	I-IV	Others	39	OS, PFS	Reported	Yes
22	Nakashima [15]	2016	124	2.4	III, IV	Pharynx	47	OS, DFS	Reported	Yes
23	Wong [17]	2016	140	3.1	I-IV	Larynx	41	OS, DFS	Reported	Yes
24	Zeng [44]	2016	115	3	III, IV	Larynx	45	OS, PFS	Reported	Yes
25	Turri_Zanoni [45]	2017	215	5.56		Others	51	OS, DFS	Reported	Yes

*NLR* neutrophil to lymphocyte ratio, *PLR* platelet to lymphocyte ratio, *OS* overall survival, *DFS* disease free survival, *PFS* progression free survival

^a^include following tumor subsite; nasopharynx, oropharynx and hypopharynx

^b^include following tumor subsite; nasal cavity or not specified

^c^A paper by Charles et al. had clinical data with two groups; oropharyngeal cancer and non-oropharyngeal cancer. We divided the results into two independent sets and employed these results separately into our analyses, named as Charles (1) and Charles (2)

### Prognostic significance of pretreatment NLR values

**Fig. 2:**
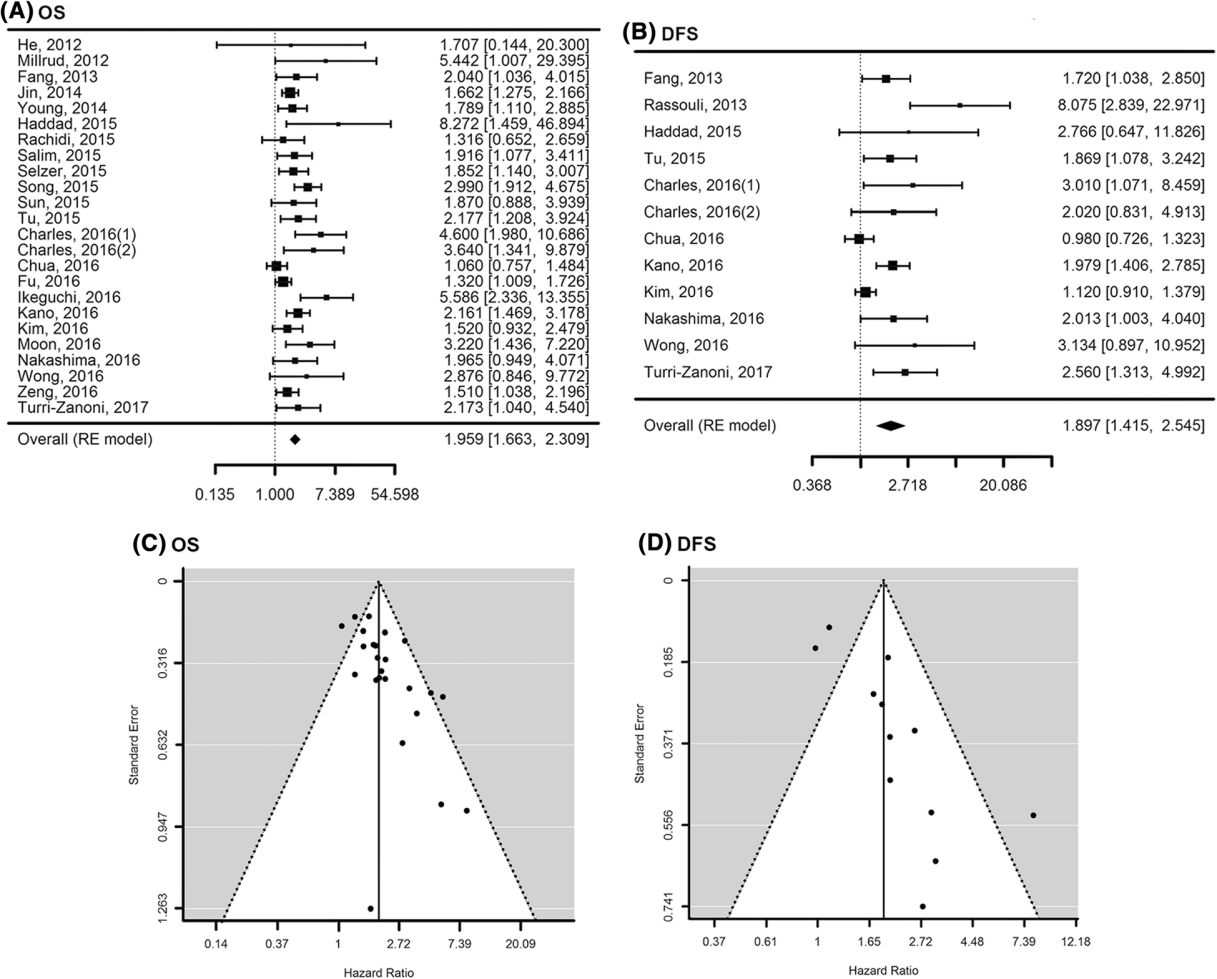
Forest plots illustrating prognostic significance of NLR value on overall survival **a** and disease-free survival **b** in HNSCC patients (The first author name of published article, Publication year). Numbers indicated the hazard ratios (HR) of survival outcomes with [95% confidence interval]. HR > 1 indicated worse outcome for the group having NLR above cutoffs compared to the group having NLR below cutoffs. OS: Overall survival, DFS: Disease-free survival, RE model: Random effect model. **c** Funnel plots of analyzed studies according to overall and disease-free survivals (**d**)

Twenty four eligible studies were analyzed in OS meta-analysis. The total HR of the random effect model was 1.96 [95%CI: 1.66–2.31] (*P* < 0.001) (Fig. 2). However, substantial heterogeneities across these studies were noted (I^2^ = 48.29%, *P* = 0.0053). Regarding DFS meta-analysis, 12 studies were enrolled. An overall HR was 1.90 [95%CI: 1.41–2.54] (*P* < 0.0001), indicating that high NLR value above cutoff was a significant predictor for DFS in HNSCC patients. The heterogeneity of these studies was also significant (I^2^ = 70.4%, *P* = 0.0002).

In terms of outcomes for PFS, there were only five studies describing PFS in their reports. An overall HR for PFS was 1.82 [95%CI: 1.43–2.33] (*P* < 0.0001), with low heterogeneity (I^2^ = 0.0%, *P* = 0.92) (Additional file 3: Figure S1). Thus, high NLR value (above cutoffs) could predict disease progression in metastatic settings of HNSCC, as well as OS and DFS. Because the number of studies was small (*n* = 5) with an outcome of PFS, we mainly focused on OS and DFS in subsequent analyses.

### Evaluation of publication bias and sensitivity analysis

Next, we evaluated publication bias by Egger’s regression test for funnel plot asymmetry. In OS meta-analysis for enrolled studies, the funnel plot showed a relatively asymmetric distribution with Egger’s *P* value of 0.0007. Similarly, there was a significant publication bias in DFS meta-analysis (*P* = 0.0017) (Fig. 2). However, a trim-and-fill method, in which we calculated the overall HR by making additional data set symmetrical to y-axis according to the midpoint of funnel plot, did not reverse results of the random effect model, confirming that there was no significant difference in the outcome (Table 2, Additional file 4: Figure S2).

**Table 2:** Results of sensitivity analysis

NLR values			Random effects model		Heterogeneity		Egger’s regression test for funnel plot asymmetry
Outcomes	Method	No. of articles	Hazard ratio (95% CI)	*P*-value	*P*-value	I squared (%)	*P*-value
OS	Raw	24	1.96 (1.66–2.31)	< 0.0001	0.0053	48.29%	0.0007
	Trim-and-fill	33	1.63 (1.35–1.97)	< 0.0001	< 0.0001	65.38%	0.5874
DFS	Raw	12	1.90 (1.41–2.54)	< 0.0001	0.0002	70.39%	0.0017
	Trim-and-fill	17	1.53 (1.12–2.08)	0.0068	< 0.0001	75.53%	0.2780

*OS* overall survival, *DFS* disease free survival, *CI* confidence interval.

### Meta-regression of cutoff values of NLR

**Fig. 3:**
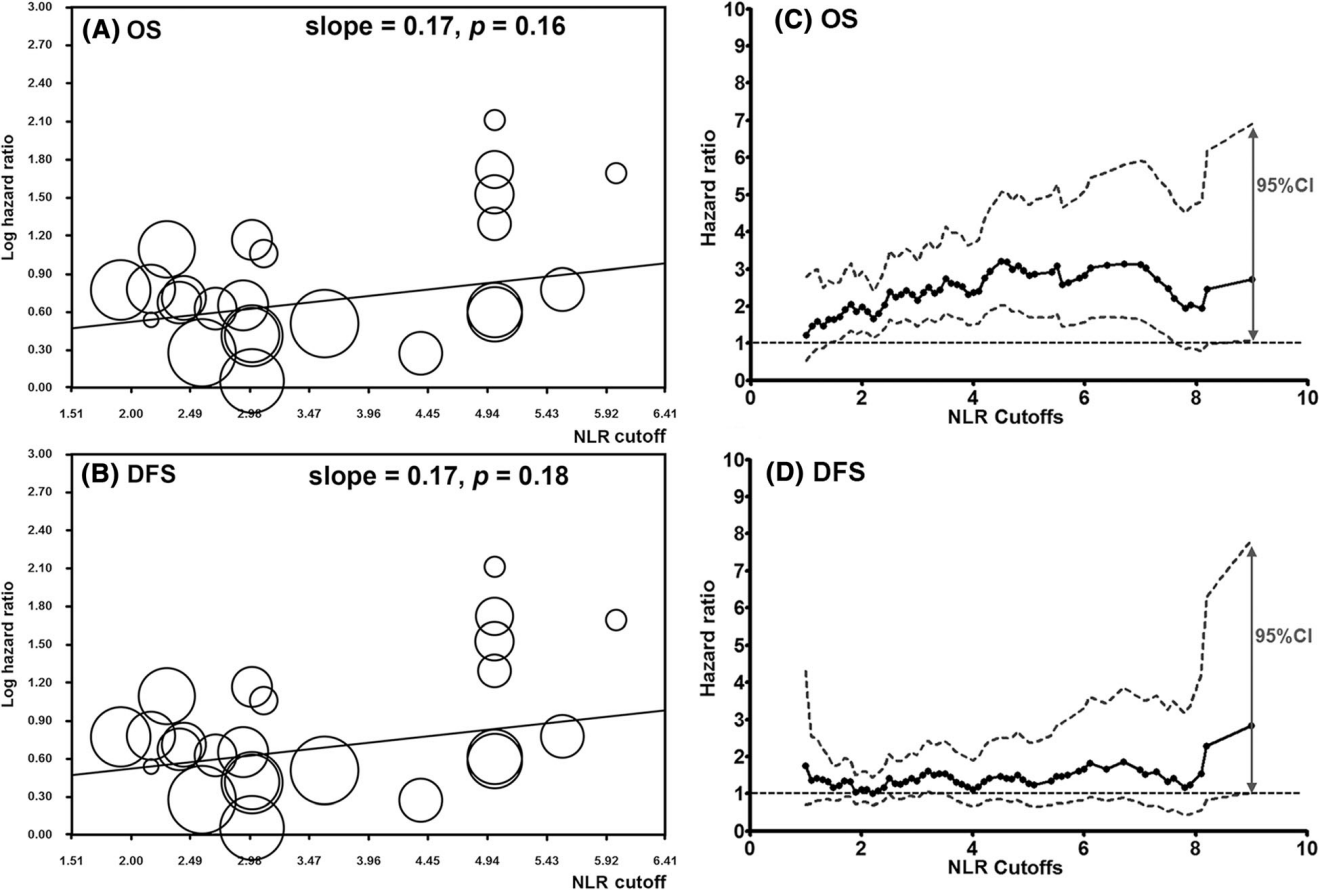
Meta-regression plots of NLR hazard ratio according to NLR cutoffs. **a** OS, **b** DFS. The center and radius of each circle indicated the Log(HR) and 95% CI of enrolled studies. X-axis meant the study-specific NLR cutoff values. **c**-**d** Hazard ratio (HR) and 95% CI according to NLR cutoffs by multivariate Cox proportional hazard regression model in an independent HNSCC cohort. **c** OS, **d** DFS. Solid line: HR, Dashed line: 95% CI. HR > 1 and 95% CI > 1 indicated significant worse outcome for the group having NLR above cutoffs compared to the group having NLR below cutoffs

Through the meta-analyses described above, we confirmed the prognostic significance of pretreatment NLR status for OS and DFS in HNSCC. However, cutoff values of NLR for dividing HNSCC patients into high and low NLR groups, ranged from 1.9 to 6.0 in enrolled studies. Thus, we next determined whether the effect of NLR status on clinical outcomes was different according to the cutoff value of NLR. To address this, we applied meta-regression for the association between the HRs of OS (or DFS) and study-specific NLR cutoff values (Fig. 3).

NLR cutoff values of 1.9 to 6.0 did not affect the extracted HRs of OS or DFS (*P* = 0.16 for OS; *P* = 0.18 for DFS). In short, absolute NLR cutoff values between 1.9 and 6.0 did not seem to matter, although groups below and above NLR cutoffs did show significant survival differences. Among other potential factors, only age variable had influenced the HRs of NLR status in OS and DFS (Additional file 1: Table S4). A prognostic significance of NLR status did not differ according to tumor subsites in the head and neck, in addition to gender, tumor stage, and multiplicity.

### A validation study using an independent data set

Next, we conducted an external validation study. For this, we used 540 registered HNSCC patients in our institute. In the independent cohort, we evaluated the prognostic impact of NLR status according to NLR cutoffs in the range between 2.0 and 6.0 by increment of 0.1. The HRs of OS were significant in any NLR cutoff values between 2.0 and 6.0 (to 7.8) (*P* < 0.001) after adjusting for age, gender, TNM stage and tumor site (a multivariate Cox hazard regression model), in line with results of the previous meta-regression analyses (Fig. 3). However, the HRs of DFS were not significant in NLR cutoffs from 1.5 to 8.5 in our cohort (*P* = 0.089).

**Fig. 4:**
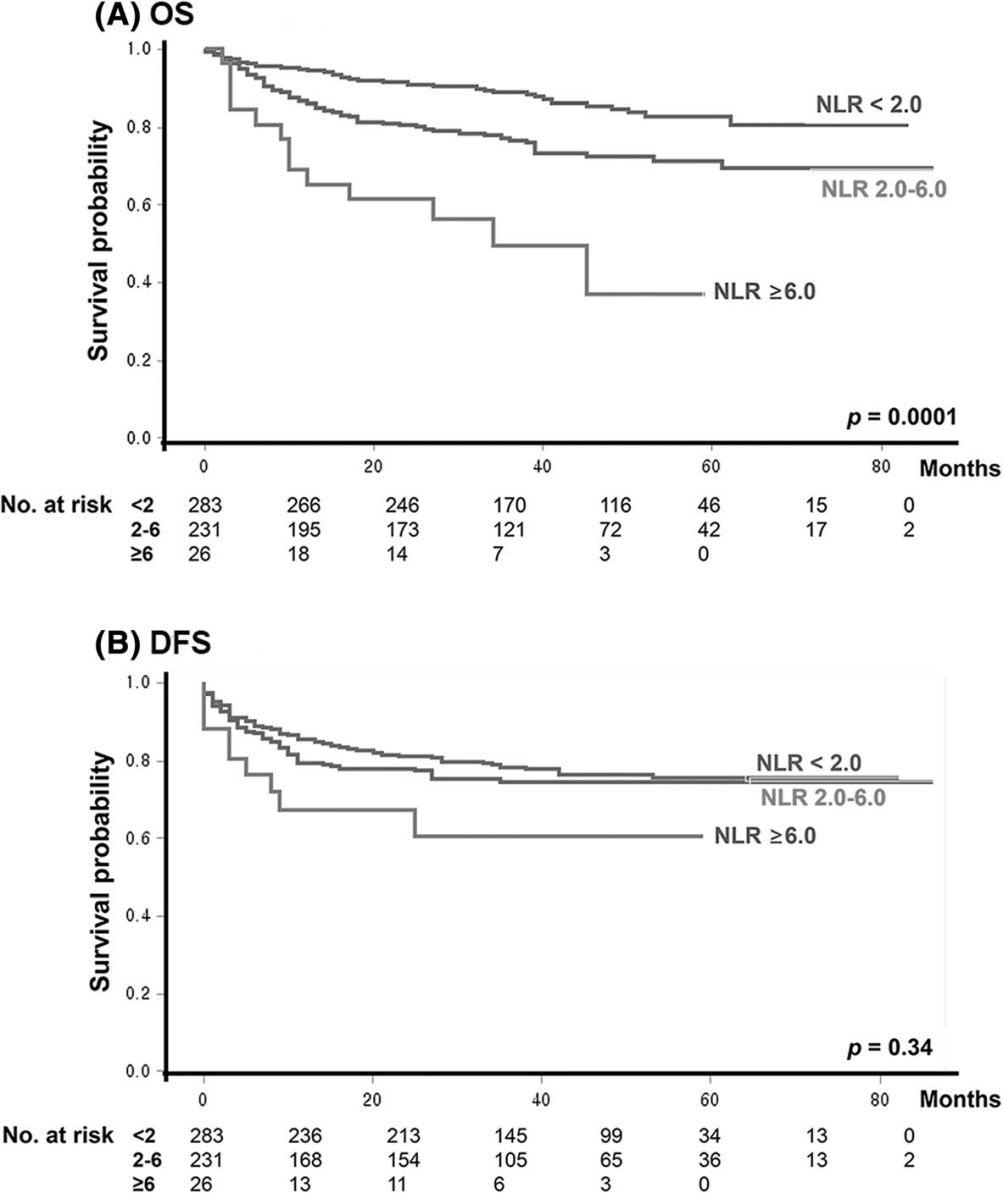
Survival difference among groups of below NLR 2.0, NLR 2 to 6 and ≥ NLR 6.0 in a validation cohort. **a** OS, **b** DFS

In other words, any NLR cutoff between 2.0 and 6.0 produced significant discrimination for better and worse prognosis group in terms of OS. For a more practical application of NLR status on prognosis estimation in HNSCC patients, our data suggested a three-tier classification of NLR status (NLR: < 2.0, 2.0–6.0, ≥ 6.0), instead of binary NLR grouping based on a single NLR cutoff. Our results also confirmed a significant discrimination of OS among groups of below NLR 2.0 (reference), NLR 2.0 to 6.0 (HR = 1.80, [95% CI: 1.13–2.86]) and above NLR ≥ 6.0 (HR = 3.92, [95% CI: 1.89–8.12]) in our cohort by a multivariate Cox proportional hazard model (adjusting for age, gender, TNM stage and subsite) (Fig. 4).

## Discussion

Our meta-analyses confirmed the prognostic impact of pretreatment NLR value in HNSCC patients. NLR is a readily measurable indicator that can be obtained from patient blood at diagnosis. Thus, it could be easily incorporated into prognosis grouping in HNSCC patients. However, its universal application is hindered by different NLR cutoffs across studies. Most studies have adopted their own cutoff values of NLR driven by internal prognosis grouping without external validation [14–19]. Thus, we conducted this study to investigate universal optimal cutoff value of NLR for consistent differentiation of HNSCC prognosis.

NLR cutoff values in the published articles have been variable among published articles, ranging from 1.9 to 6.0. Interestingly, our meta-regression analysis revealed that NLR status according to any NLR cutoffs between 1.9 and 6.0 had similar prognostic impact on OS and DFS. In a validation cohort, we observed consistent results in terms of OS outcome. Thus, it is hard to determine a single specific cutoff value between 2.0 and 6.0 as a NLR cutoff for binary OS prognosis grouping. Rather, it indirectly suggests a bimodal distribution of patients (i.e., large proportions of patients with better prognosis below NLR = 2.0 and large portion of patients with worse prognosis above NLR = 6.0). As a practical conclusion with prognostic impact of pretreatment NLR on HNSCC patients, our results showed that three-tier categorization (NLR values < 2.0, 2.0 to 6.0, ≥ 6.0) would be more clinically relevant and easy to be translated it to clinics.

One thing to note was that our results from this meta-analysis were not validated in our cohort in terms of DFS outcomes. Reasons for this discrepancy include different distribution of HNSCC subsites, treatment-related factors and patient factors. Systemic inflammation may reflect host response to cancer associated inflammation or immune reaction [1, 2, 4]. Thus, it is reasonable to think that NLR status might be more related to patient overall outcomes, than to local tumor control. For example, dysfunctional larynx due to laryngeal cancer or treatments can cause patient mortality (aspiration pneumonia) without metastatic disease. In such case, we frequently observe elevated inflammatory markers (for example, C-reactive protein). However, this assumption needs to be investigated further in the future studies.

Although we employed a statistical method of meta-analysis and performed validation with an independent cohort, our study still had some limitations to draw a solid conclusion. First, there were the heterogeneity issue and publication bias of articles included in our meta-analyses. A significant heterogeneity across these studies was found in both DFS and OS meta-analyses except for PFS analysis. Similarly, there was a significant publication bias in DFS and OS meta-analysis. Thus, these enrolled studies seemed not to be representative of NLR significance in HNSCC, and the positive results of NLR values on prognostic impacts might be published more in the literature than the negative results of NLR values. To minimize bias, we adopted a random effect model to estimate the overall HR of DFS and OS and performed a sensitivity analysis (trim-and-fill method) to confirm our primary analyses in this study. Nevertheless, the further studies with less heterogeneity and publication bias are needed to confirm our conclusion.

Head and neck cancers include a group of cancers arising from the various sites in the head and neck. In this study, we employed multivariate analyses adjusting tumor subsites in the meta-regression analysis (Additional file 1: Table S4) and in the validation set (Fig. 4), and confirmed that a prognostic significance of NLR status did not differ according to tumor subsites in the head and neck. However, the validation cohort did not include the patients with nasopharyngeal cancer (Additional file 1: Table S3). Thus, our results should be re-evaluated with a site-specific prospective cohort of head and neck cancers. In addition, enrolled studies in meta-analyses did not report the human papilloma virus (HPV) or p16 status in HNSCC. Thus, we could not analyze the significance of NLR values according to the HPV status of HNSCC in this study.

## Conclusions

Pretreatment high NLR values above the cutoff were significantly associated with shorter survival in HNSCC patients. NLR values below two and above six could consistently differentiate better and worse prognosis in HNSCC patients, which might be readily translated to clinics.

## Additional files

Additional file 1: **Table S1.** Searching strategy. **Table S2.** Quality assessment of included studies (Newcastle-Ottawa scale). **Table S3.** Clinical characteristics of a validation cohort (*n* = 540). **Table S4.** Meta-regression models for hazard ratios (HR) of NLR status. (DOCX 26 kb)

Additional file 2: Raw data for validation study. (XLSX 71 kb)

Additional file 3: **Figure S1.** Prognostic significance of NLR value on progression-free survival (PFS) in HNSCC patients (The first author name of published article, Publication year). (A) Forest plot, (B) Funnel plot. Numbers indicated the hazard ratios (HR) of survival outcomes with [95% confidence interval]. HR > 1 indicated worse outcome for the group having NLR above cutoffs compared to the group having NLR below cutoffs. RE model: Random effect model. (TIF 2385 kb)

Additional file 4: **Figure S2**. A sensitivity analysis to adjust publication bias. (A) OS, (B) DFS. (TIF 3062 kb)
