# Supplementary material for: Optimal cutoff of pretreatment neutrophil-to-lymphocyte ratio in head and neck cancer patients: a meta-analysis and validation study
Source: BMC Cancer. 2018 Oct 11;18:969. doi: 10.1186/s12885-018-4876-6 (PMC6182814; doi:10.1186/s12885-018-4876-6)
Supplement: Supplementary file 1 — Table S1. Searching strategy. Table S2. Quality assessment of included studies (Newcastle-Ottawa scale). Table S3. Clinical characteristics of a validation cohort (n = 540). Table S4. Meta-regression models for hazard ratios (HR) of NLR status. (DOCX 26 kb) [file 12885_2018_4876_MOESM1_ESM.docx]

**Table S1.** Searching strategy

| **Database: Medline, Embase and Cochrane library <0000 to April 30^th^. 2017>** | |
| --- | --- |
|  | **Cancer** |
| 1 | Neoplasms |
| 2 | Cancer* OR Tumor* OR Tumour* OR carcinoma* OR neoplasm* OR malignant* |
| 3 | Squamous* OR “squamous cell” OR “squamous cell carcinoma” OR SCC |
| 4 | #1 OR #2 OR #3 |
|  | **Head and Neck** |
| 5 | Oral or mouth |
| 6 | Nasopharynx* |
| 7 | Oropharynx* |
| 8 | Hypopharynx* |
| 9 | Larynx* |
| 10 | Pharynx* |
| 11 | “nasal cavity” OR nasal |
| 12 | #5 OR #6 OR #7 OR #8 OR #9 OR #10 OR #11 |
|  | **Neutrophil lymphocyte ratio** |
| 13 | Neutrophil* |
| 14 | NLR |
| 15 | “neutrophil to lymphocyte” OR “neutrophil to lymphocyte ratio” |
| 16 | #13 OR #14 OR #15 |
|  | **Prognosis** |
| 17 | Prognosis OR prognos* |
| 18 | Survival OR “survival analysis” OR “survival analyses” |
| 19 | #17 OR #18 |
|  | **Combined results** |
|  | #4 AND #12 AND #16 AND #19 |

**Table S2.** Quality assessment of included studies (Newcastle-Ottawa scale)

| **Study** | **Selection** | | | | **Comparability** | | **Exposure** | | | **Score** |
| --- | --- | --- | --- | --- | --- | --- | --- | --- | --- | --- |
|  | **Case-cohort representative** | **Selection of non-exposed control** | **Ascertainment of exposure** | **Outcome negative at start** | **Comparability by design** | **Comparability by analysis** | **Outcome assessment** | **Duration of follow-up** | **Adequacy of follow-up** |  |
| He(24) | **★** | **★** | **★** | **★** | **★** | **★** | **★** | **★** | **★** | 9 |
| Millrud(25) | **★** | **★** | **★** | **★** | **★** |  | **★** | **★** | **★** | 8 |
| Fang(26) | **★** | **★** | **★** | **★** | **★** |  | **★** | **★** | **★** | 8 |
| Rassouli(18) | **★** | **★** | **★** | **★** | **★** |  | **★** | **★** | **★** | 8 |
| Jin(27) | **★** | **★** | **★** | **★** |  |  | **★** | **★** | **★** | 7 |
| Young(28) | **★** | **★** | **★** | **★** |  | **★** | **★** | **★** | **★** | 8 |
| Haddad(29) | **★** | **★** | **★** | **★** | **★** |  | **★** | **★** | **★** | 8 |
| Rachidi(30) | **★** | **★** | **★** | **★** |  | **★** | **★** | **★** | **★** | 8 |
| Salim(31) | **★** | **★** | **★** | **★** |  |  | **★** | **★** | **★** | 7 |
| Selzer(32) | **★** | **★** | **★** | **★** |  | **★** | **★** | **★** | **★** | 8 |
| Song(33) | **★** | **★** | **★** | **★** | **★** |  | **★** | **★** | **★** | 8 |
| Sun(19) | **★** | **★** | **★** | **★** |  | **★** | **★** | **★** | **★** | 8 |
| Tu(16) | **★** | **★** | **★** | **★** | **★** | **★** | **★** | **★** | **★** | 9 |
| Charles(14) | **★** | **★** | **★** | **★** | **★** | **★** | **★** | **★** | **★** | 9 |
| Chua(34) | **★** | **★** | **★** | **★** | **★** | **★** | **★** | **★** | **★** | 9 |
| Fu(35) | **★** | **★** | **★** | **★** |  | **★** | **★** | **★** | **★** | 8 |
| Ikeguchi(36) | **★** | **★** | **★** | **★** |  | **★** | **★** | **★** | **★** | 8 |
| Kano(37) | **★** | **★** | **★** | **★** |  | **★** | **★** | **★** | **★** | 8 |
| Kim(38) | **★** | **★** | **★** | **★** |  | **★** | **★** | **★** | **★** | 8 |
| Moon(39) | **★** | **★** | **★** | **★** |  | **★** | **★** | **★** | **★** | 8 |
| Nakashima(35) | **★** | **★** | **★** | **★** | **★** |  | **★** | **★** | **★** | 8 |
| Wong(17) | **★** | **★** | **★** | **★** | **★** | **★** | **★** | **★** | **★** | 9 |
| Zeng(40) | **★** | **★** | **★** | **★** | **★** | **★** | **★** | **★** | **★** | 9 |
| Turri_Zanoni(41) | **★** | **★** | **★** | **★** |  | **★** | **★** | **★** | **★** | 8 |

**Table S3.** Clinical characteristics of a validation cohort (*n* = 540)

| **Clinical characteristics** | | | | **Number (%)** |
| --- | --- | --- | --- | --- |
| Gender (M:F) | | | | 422:118 (78.1:21.9) |
| Age (Mean, SD, years) | | | | 59.7±11.7 |
| Neutrophil-lymphocyte ratio (Mean, SD, range) | | | | 2.4±1.9 (0.38-18.5) |
| Primary sites | | | |  |
|  | | | Oral cavity | 214 (39.6) |
|  | | | Larynx | 152 (28.1) |
|  | | | Pharynx (Oro & Hypopharynx) | 119 (22.0) |
|  | | | Others | 55 (10.2) |
| TNM stage | | | |  |
|  | | | I | 215 (39.8) |
|  | | | II | 69 (12.8) |
|  | | | III | 68 (12.6) |
|  | | | IV | 188 (34.8) |
| Treatment outcomes | | | |  |
|  | | No disease recurrence | | 415 (76.9) |
|  | | Local recurrence | | 41 (7.6) |
|  | | Regional recurrence | | 35 (6.5) |
|  | | Loco-regional recurrence | | 10 (1.9) |
|  | | Distant metastasis | | 29 (5.4) |
|  | | Residual disease | | 10 (1.9) |
| Patient status at last follow-up | | | |  |
|  | No evidence of disease | | | 404 (74.8) |
|  | Alive with disease | | | 18 (3.3) |
|  | Death of disease | | | 84 (15.6) |
|  | Death of other diseases | | | 11 (2.0) |
|  | Unknown | | | 23 (4.3) |

**Table S4.** Meta-regression models for hazard ratios (HR) of NLR status

| **HR of NLR status** | **Overall survival** | |  | **Disease-free survival** | |
| --- | --- | --- | --- | --- | --- |
|  | **R^2^** | **Test of moderators**  **(*P*-value)** |  | **R^2^** | **Test of moderators**  **(*P*-value)** |
| Cutoff values of NLR | 0.00% | 0.1606 |  | 4.86% | 0.1756 |
| Age | 23.58% | 0.0171 |  | 58.88% | 0.0080 |
| Gender (M/F) | 0.00% | 0.7636 |  | 3.80% | 0.3111 |
| TNM stage (III, IV/I, II) | 0.00% | 0.9575 |  | 0.00% | 0.8645 |
| Tumor sites  (Oral cavity/ Pharynx/ Larynx/ Others) | 0.00% | 0.7645 |  | 0.00% | 0.9164 |
| Tumor multiplicity (Multiple/ Single) | 0.00% | 0.5626 |  | 0.00% | 0.7472 |

R^2^ : Explanatory power of the regression model (0-100%)
