# Supplementary figures and images for: Optimal cutoff of pretreatment neutrophil-to-lymphocyte ratio in head and neck cancer patients: a meta-analysis and validation study
Source: BMC Cancer. 2018 Oct 11;18:969. doi: 10.1186/s12885-018-4876-6 (PMC6182814; doi:10.1186/s12885-018-4876-6)

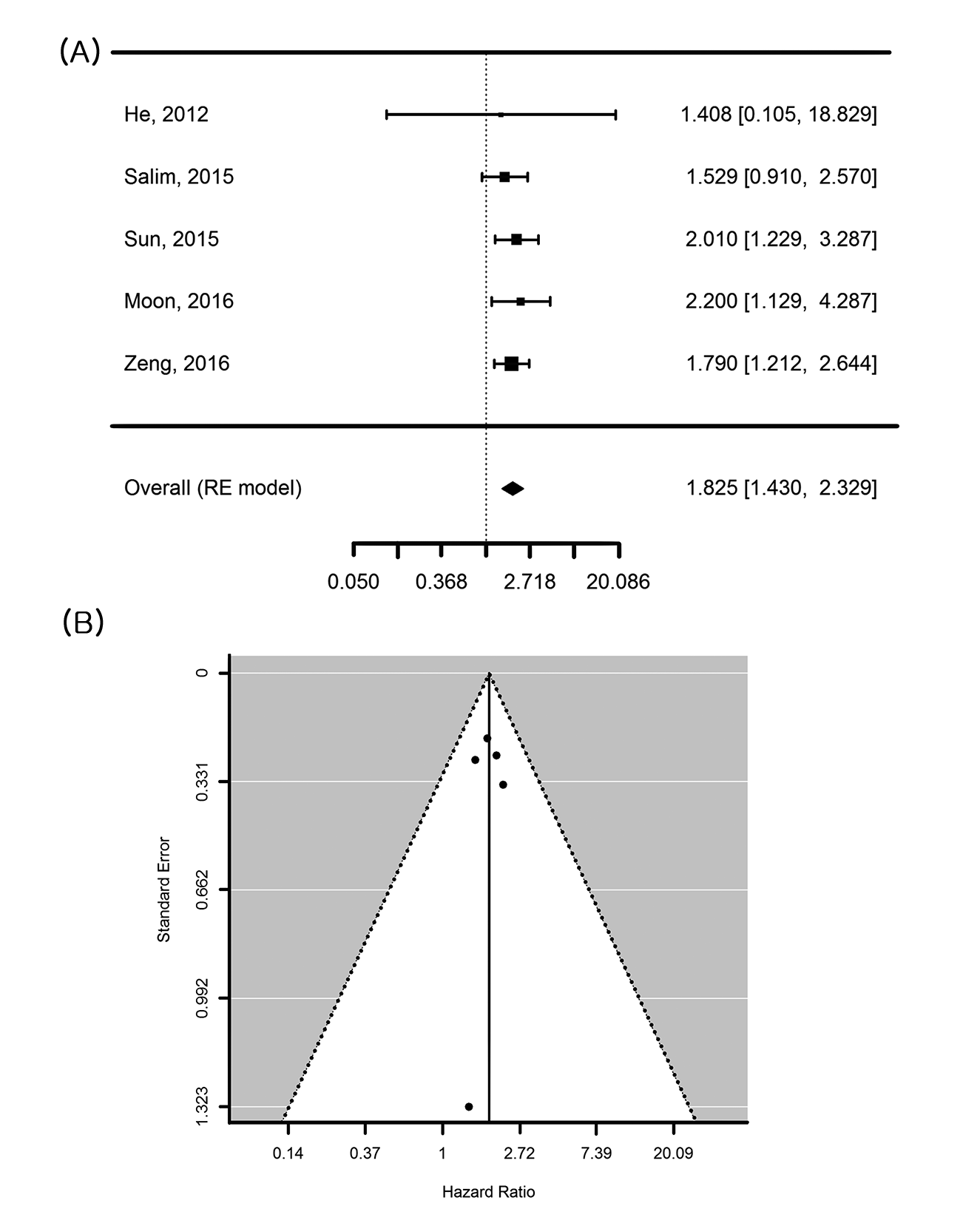

Supplement: Supplementary file 3 — Figure S1. Prognostic significance of NLR value on progression-free survival (PFS) in HNSCC patients (The first author name of published article, Publication year). (A) Forest plot, (B) Funnel plot. Numbers indicated the hazard ratios (HR) of survival outcomes with [95% confidence interval]. HR > 1 indicated worse outcome for the group having NLR above cutoffs compared to the group having NLR below cutoffs. RE model: Random effect model. (TIF 2385 kb) [file 12885_2018_4876_MOESM3_ESM.tif]

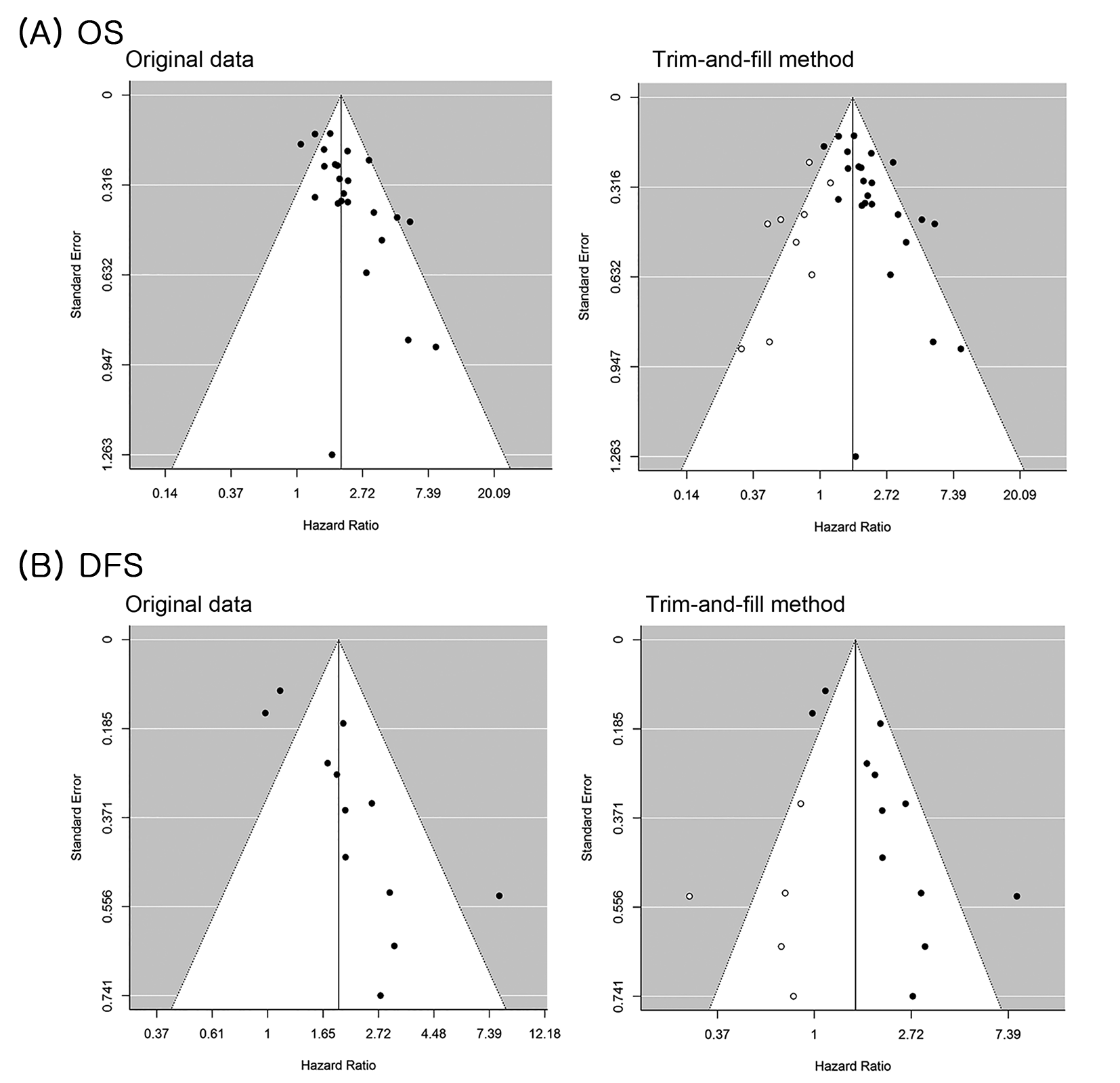

Supplement: Supplementary file 4 — Figure S2. A sensitivity analysis to adjust publication bias. (A) OS, (B) DFS. (TIF 3062 kb) [file 12885_2018_4876_MOESM4_ESM.tif]
